# Bio-Inspired Vision and Gesture-Based Robot-Robot Interaction for Human-Cooperative Package Delivery

**DOI:** 10.3389/frobt.2022.915884

**Published:** 2022-07-07

**Authors:** Kaustubh Joshi, Abhra Roy Chowdhury

**Affiliations:** ^1^ Department of Mechanical Engineering, University of Maryland, College Park, MD, United States; ^2^ Centre for Product Design and Manufacturing, Division of Mechanical Engineering, Indian Institute of Science (IISc), Bangalore, India

**Keywords:** bio-inspired multi-robot interaction, RGB-D perception, vision based gestural interaction, human-robot cooperation, passive action recognition

## Abstract

This research presents a novel bio-inspired framework for two robots interacting together for a cooperative package delivery task with a human-in the-loop. It contributes to eliminating the need for network-based robot-robot interaction in constrained environments. An individual robot is instructed to move in specific shapes with a particular orientation at a certain speed for the other robot to infer using object detection (custom YOLOv4) and depth perception. The shape is identified by calculating the area occupied by the detected polygonal route. A metric for the area’s extent is calculated and empirically used to assign regions for specific shapes and gives an overall accuracy of 93.3% in simulations and 90% in a physical setup. Additionally, gestures are analyzed for their accuracy of intended direction, distance, and the target coordinates in the map. The system gives an average positional RMSE of 0.349 in simulation and 0.461 in a physical experiment. A video demonstration of the problem statement along with the simulations and experiments for real world applications has been given here and in Supplementary Material.

## 1 Introduction

**FIGURE 1:**
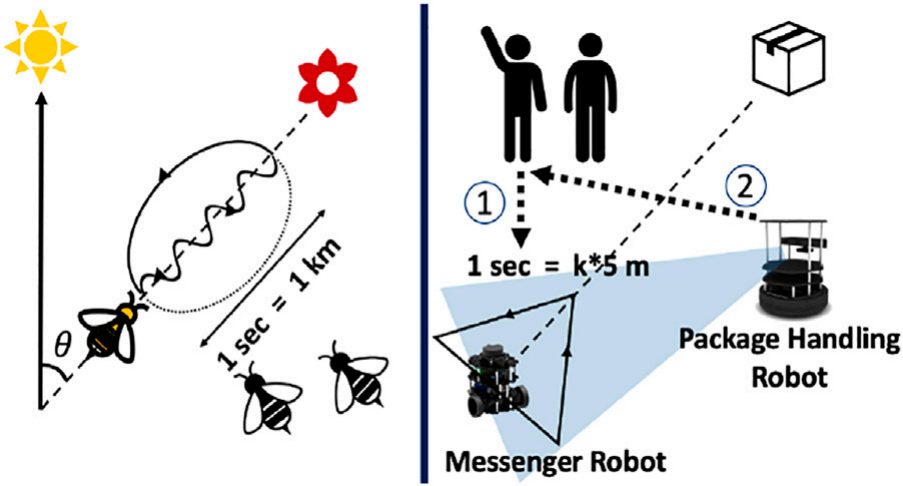
An analogical example of how bees communicate to convey distance and direction of food source. The framework used in this paper involves a robot that moves in a triangle to convey direction and distance towards the target destination for the other robot.in cooperation with humans.

Humans are adept at using audio and visual cues for communication while carrying out collaborative tasks. However, humans must rely entirely on non-verbal communication like visual gestures to coordinate in a noisy environment. This research aims towards implementing a similar ability to use gestural interaction in a networked system of robots. Traditional methods of robot communication in a multi-robot system rely heavily on network connections through communication protocols (Yan et al., 2013). What if a system of robots had to be deployed in an area where there was a lack of network resources? In such a scenario, robots will have to rely on other sensors for interaction. In this paper, two robots are used to demonstrate a vision-based gestural interaction framework to carry out a task of package handling in cooperation with a human (Wang and Schwager, 2015). This approach enables each robot to be independent of a centralized controller or server. A system of two different robots demonstrates that the framework is scalable to incorporate different types of robots in the system. The system consists of the following types of robots: (1) package handling robots capable of carrying packages and detecting both human and robot gestures; (2) messenger robots for conveying information to package handling robots and carry out supervision and can detect only human gestures. The messenger robots read a human gesture using skeletal tracking for knowing the package to be transported and signal a particular package handling robot to carry out the task. The package handling robot is capable of object detection and tracking by which it can infer the trajectory of a messenger robot depicting a particular action. This mode of bio-inspired passive action recognition (Das et al., 2016) is based on how bees communicate with other bees in their hive to convey the distance and direction of a food source about the hive’s location and position of the Sun using a gesture-based communication called ‘waggle dance’ (Riley et al., 2005) (See Figure 1). Waggle dance has been used in for studying pattern formation and recognition (Esch and Burns, 1996). Recent studies have used it for robot interaction with live bees (Landgraf et al., 2018). In multi-robot systems, “waggle dance” inspired interaction has been used to identify commands in the form of gestures from other robots (Das et al., 2016). This paper implements a novel framework for interaction as well as determining the distance and direction through such gestures. Past work in vision-based multi robot interaction (Kuniyoshi et al., 1994) has mainly focused on limited explicit communication (Meng et al., 2006) and marker-based communication (Parker et al., 2004) or colour-based interaction (Melanie et al., 2020). This research adds a gestural interaction framework to the existing literature. Gesture based interaction involves object detection and tracking for which various algorithms have been developed in the past. The major object detection algorithms include the development of YOLO (You Only Look Once) (Redmon et al., 2016), (Shafiee et al., 2017), (Redmon and Farhadi, 2018) series as well as SSD (Single Shot Detection) (Liu et al., 2016), (Zhai et al., 2020) frameworks. Object tracking involves the usage of measuring the distance of that particular object from the camera. This has been done using a monocular camera (Crivellaro et al., 2017), (Tjaden et al., 2018) using machine learning algorithms, or a stereo camera (Lin and Wang, 2010), (Issac et al., 2016) using triangulation methods as well as other machine learning methodologies. The current paper uses a depth camera (Lukezic et al., 2019) where the IR sensor in the depth camera is used to measure the distance of the object from the camera once it has been detected. Object tracking has various methodologies being used in the past like centroid detection and tracking (Nascimento et al., 1999), average depth estimation (Chu et al., 2017) and other methods.

Additionally, network servers are prone to damage by heat, moisture and dust. Moreover, a lack of electricity or network is possible during natural calamities, due to which a server may not be feasible. Hence, the approach ensures that the system is reliable and in cases of emergencies, can respond to human intervention and instruction to stop a certain process. To summarize, the contributions of this paper are as follows:

1) A distributed, asynchronous and scalable robot-robot interaction using vision-based gestural motion cues
2) Robot-robot interaction using a gesture-based action formation and recognition methodology:
a) Gesture formation in form of shapes using basic motion control
b) Gesture recognition of formed shapes using visual perception
c) Target Position Identification by formulating the orientation and duration of gesture
3) Experimental validation in simulation environment and a physical system of two robots with multiple humans on an experimental testbed of a manufacturing industry setup.

## 2 Problem Formulation

**FIGURE 2:**
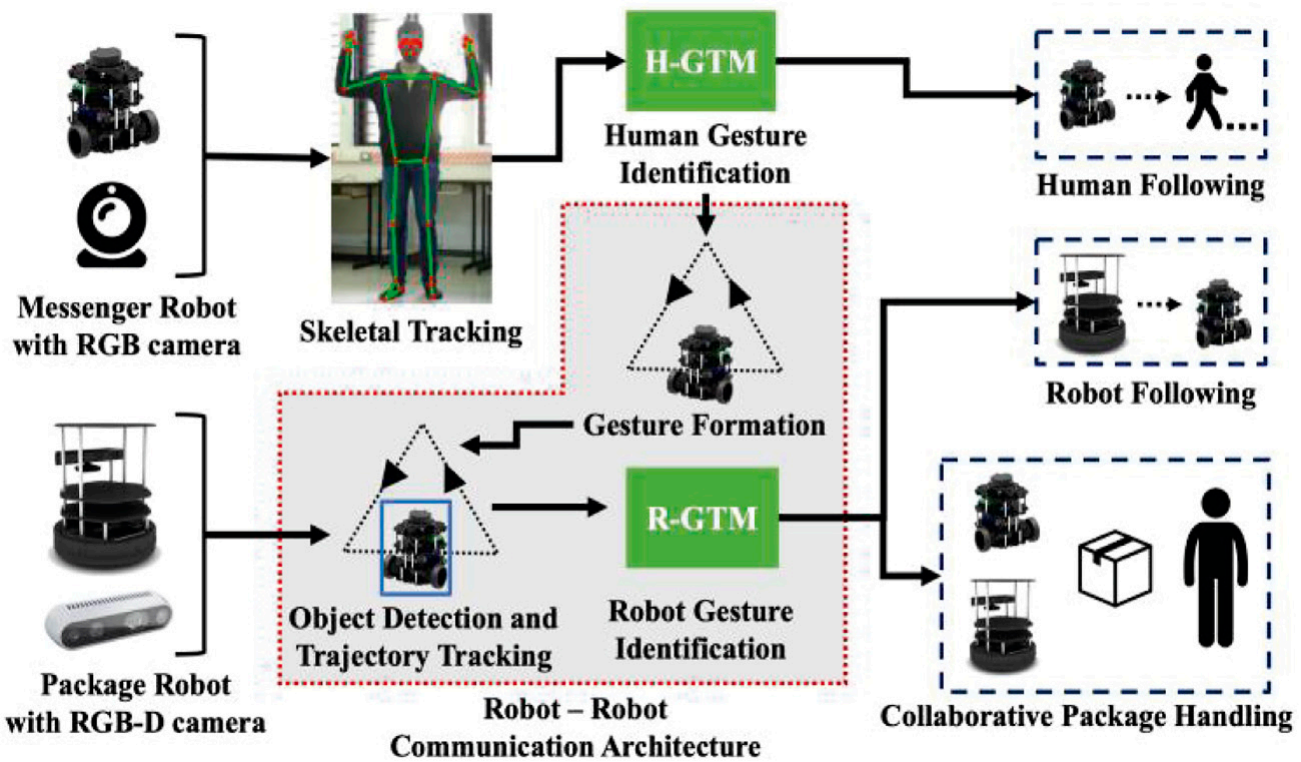
Schematic for a collaborative vision-based gestural interaction framework. The shaded region represents the contribution of this paper.

The main focus of this paper is to demonstrate a robot-robot interaction framework using vision-based cues. Additionally, this is also done in collaboration with a human to carry out a package delivery task. The main methodology is to identify the robot and track its trajectory through which a gesture is interpreted, and the command is carried out. Figure 2 provides an overview of the entire schematic of the proposed architecture.

### 2.1 Human Gesture Identification

**FIGURE 3:**
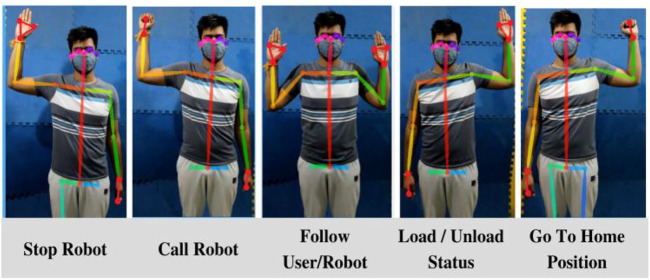
Commands for robot correlating human gestures.

This paper uses skeletal tracking for human body pose estimation to convey a command to the robot. Google’s MediaPipe (Lugaresi et al., 2019) is used for tracking the skeletal points. A total of 18 skeletal points of the upper body are taken. Based on the coordinates of these skeletal points, certain body poses are pre-coded into the robot as certain gestures with corresponding commands, as shown in Figure 3. Improving skeletal tracking and identification is not the focus of this paper. Body pose estimation is merely used to introduce a human-in-the-loop for a cooperative package handling task.

### 2.2 Robot-Robot Interaction

The system consists of the following types of robots: (1) package handling robots capable of carrying packages and detecting both human and robot gestures; (2) messenger robots for conveying information to package handling robots and can detect only human gestures. The messenger robots read a human gesture using skeletal tracking for knowing the package to be transported and signal a particular package handling robot to carry out the task. The package handling robot is capable of object detection and tracking by which it can infer the trajectory of a messenger robot depicting a particular action. The methodology concerned with gesture formation and identification are discussed exhaustively in the next section (see Section 3).

### 2.3 Path Planning and Control

The robots are given a map of the environment before-hand constructed using RTAB-MAP (Labbé and Michaud, 2019). Once they receive command with destination location, a global path planner is implemented using the A* algorithm, (Hart et al., 1968), and Dynamic Window Approach (Fox et al., 1997) is used for local path planning. A proportional controller is used on the robot to gradually approach the target location or follow a person or robot. The distance for following is calculated based on the size of the bounding box of objects in the camera frame. These methods are merely for demonstration, and better ones exist in the literature, but that is not the focus of this paper.

## 3 Robot-Robot Interaction

### 3.1 Robot Gesture Formation

Following a human command, the robot has to convey instructions to another robot. The messenger robot moves in basic shapes according to the type of command (see Table 1). As an extension to this gesture-based command transfer, a bio-inspired gesture attribute recognition, as shown in Figure 1, is demonstrated for the case of a triangle, as explained in Table 1.

**TABLE 1:** Classification of different types of shapes and their corresponding gestures.

Shape	Command	Command attributes
Triangle	Go to location	Orientation of triangle denotes the direction; Duration of gesture correlates to distance to be travelled
Circle	Follow messenger	N/A
Square	Follow a human	N/A

#### 3.1.1 Shape Formation

Before forming a gesture, the messenger robot approaches and faces the other robot head-on using pose estimation from a custom trained YOLOv4 (Bochkovskiy et al., 2020) model. Subsequently, shapes are formed by the robot discussed in Table 1 using basic Robot Operating System (ROS) commands (Quigley et al., 2009). In the case of a triangle, the robot has to initially orient itself to angle α, which will be the initial angle the robot has to turn to before starting its motion to trace a triangle in direction θ, which is the direction the robot has to convey to the other robot in the framework. To prevent large odometry errors, an angular velocity of ω_i_ = 15°/sec is chosen for the robot. An equilateral triangle is formed with sides a = 0.4 m. Larger sides have not been chosen since it leads to higher errors in odometry. Similarly, smaller a has not been chosen since the shapes formed are not distinguishable due to a smaller area. The triangles are formed at an orientation of ϕ with a constant linear speed of v and angular speed of ω_δ_ = 30°/sec. Here, ϕ is decided by the direction of the package handling robot from its current pose.

#### 3.1.2 Duration Control of Gesture

The goal here is to find a v such that the triangle is traversed in time T at an orientation of ϕ. Accordingly, to cover the trajectory after orienting itself, the relation of duration T to linear speed v of the robot is:

$$\begin{array}{l} T=3*\frac{a}{v}+3*\frac{\left(\frac{2\pi }{3}\right)}{\omega_{\delta }}\;\mathrm{secs}\\ ∴v=\frac{1.2}{T-12}m/\sec \end{array}$$ (1)

### 3.2 Robot Gesture Identification

**FIGURE 4:**
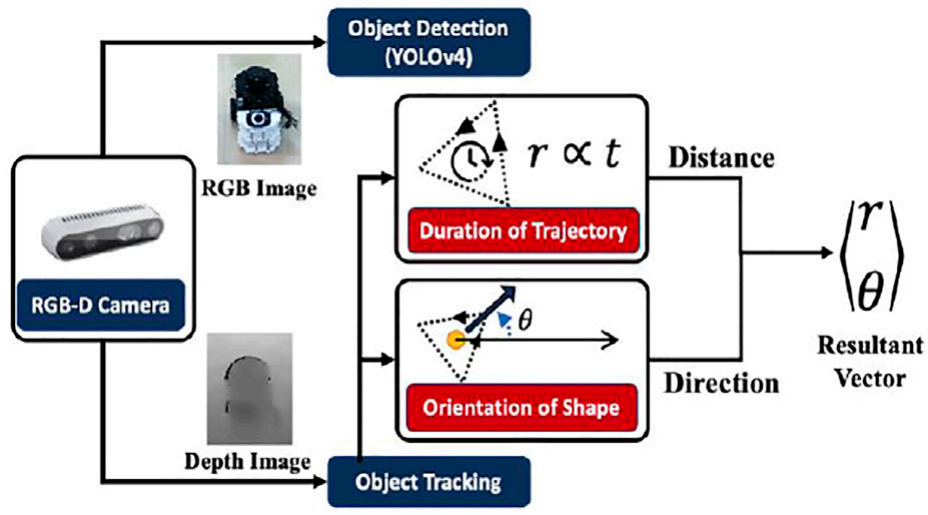
Schematic for the methodology of Gesture Identification and Inference.

After gesture formation, the package handling robot recognizes the gestures based on shape, orientation, and motion duration. The next step after gesture formation is for the package handling robot to recognize the gesture based on the shape, orientation and time in which gesture is completed. An overall schematic of the same is depicted in Figure 4.

#### 3.2.1 Object Detection

Object detection is implemented on the RGB image from the depth camera for identifying the robot. The YOLOv4 (Bochkovskiy et al., 2020) model is trained on a 500-image custom dataset. The primary aim is to identify the robot and implement a better object detection model beyond this paper’s scope.

#### 3.2.2 Object Trajectory Tracking

The depth image from the RGB-D camera is used for finding the corresponding depth of points depicted in the bounding box given by the object detection algorithm. An average of all the points is given as the distance of the robot from the camera, and the coordinates of the robots are tracked. The trajectory of the robot is tracked for its shape and time taken to complete. An important point to note in this section is that the messenger robot moves in a 2D plane but is tracked in a 3D space. This creates a discrepancy in the actual path followed by the messenger robot against the path observed by the package handling robot. However, the shape is similar when viewed from a top view.

#### 3.2.3 Gesture Recognition

The package handling robot is trained to identify the shape of trajectory in relation to the area covered using the methodology for calculating the extent of the area implemented (Das et al., 2016). Let the shape be x,y ∈ [X,Y ]. The area of the polygon has to be calculated to find the area (A).

$$A=\left|\frac{\left(\sum_{i=1}^{N}x_{i}*y_{i+1}-x_{i+1}*y_{i}\right)+\left(x_{N}*y_{1}-x_{1}*y_{N}\right)}{2}\right|$$ (2)

To classify a shape, the ratio of area to its bounding box is calculated as extent of area (Das et al., 2016). The bounding box was marked using the minimum and maximum x and y coordinates tracked by the camera. Different shapes tend to have different ranges of extent of area classified empirically after conducting various test cases.

#### 3.2.4 Target Position Identification

A gesture formation and recognition methodology is also implemented for robots to convey a specific point to visit on the map. The duration of the gesture and its orientation to another robot corresponds to the resultant vector from robot to target pose. The case of a triangle is used to depict the task of going to a particular location {*x*_*T*_ *,y*_*T*_} from the source location of robot {*x*_*0*_ *,y*_*0*_}. For calculating distance, the time is taken, **T**, to cover the path corresponds to the distance, ***r***, the robot has to travel. Here, ***r*** is to be estimated by the robot, and subsequently, *x*_*T*_ and *y*_*T*_ once the direction is known

$$r=\sqrt{{\left(x_{T}-x_{0}\right)}^{2}-{\left(y_{T}-y_{0}\right)}^{2}}$$ (3)

This distance inferred by the robot correlates to the total time taken (T) by the messenger robot to complete the gesture. As discussed earlier, a shorter duration corresponds to a shorter distance.

$$r\propto T\;\&.\;r\propto \frac{1}{v}$$ (4)

Assuming that the maximum speed of the robot is *v*_*max*_ and maximum traversal distance is *d*_*max*_

$${r|}_{d_{\max}}={k*\left(\frac{3*a}{v}+3*\frac{\pi }{3*\omega }\right)|}_{v_{\max}}$$ (5)

Where, where a=0.4m is the length of side of triangle, v is the linear speed of the robot, ω=π/12 is the angular velocity of the robot and k is the proportionality constant with which the entire time period required is multiplied. Formulating Eq. 5, we get

$$k=\frac{d_{\max}*v_{\max}}{12*\left(0.1+v_{\max}\right)}$$ (6)

Hence, the relation of distance with time duration of gesture is

$$r=\frac{d_{\max}*v_{\max}}{12*\left(0.1+v_{\max}\right)}*T$$ (7)

Calculating Direction: The perpendicular to the vertex of the triangle where the gesture starts/ends, from the opposite base denotes the target direction, and the centroid of the triangle lies on its perpendicular. Let {x_obs_,y_obs_} ∈ C, where C is the set of observed coordinates of robot detected by the camera. The centroid {x_ctd_,y_ctd_} is

$$\left\{x_{\mathrm{ctd}},y_{\mathrm{ctd}}\right\}=\left\{\frac{\sum_{0}^{N-1}x_{\mathrm{obsi}}}{N},\frac{\sum_{0}^{N-1}y_{\mathrm{obsi}}}{N}\right\}$$ (8)

The coordinates of the vertex are taken by taking an average of the first 5, and the last 5 points in C denoted as {x_vtx_,y_vtx_}. Therefore, the resultant angle of direction is formulated as

$$\theta \;=\;\mathrm{atan}2\left(y_{\mathrm{vtx}}-y_{\mathrm{ctd}},x_{\mathrm{vtx}}-x_{\mathrm{ctd}}\right)$$ (9)

Thus, we get a resultant polar vector of { $\vec{r}$ ,θ} towards the target location, which is translated to cartesian coordinates on the map as the target pose for the robot.

## 4 Experiment Set-Up

**FIGURE 5:**
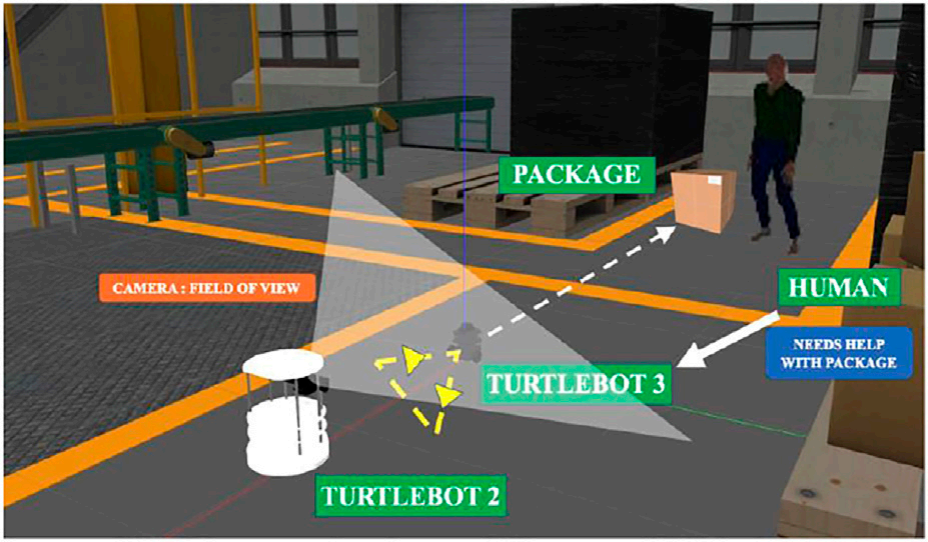
Simulation Environment.

The simulation environment depicted in Figure 5 is created in the Gazebo (Koenig and Howard, 2004) simulator. The robots used are a Turtlebot 2 equipped with a Kinect camera at the height of 90 cm and a Turtlebot 3.

**FIGURE 6:**
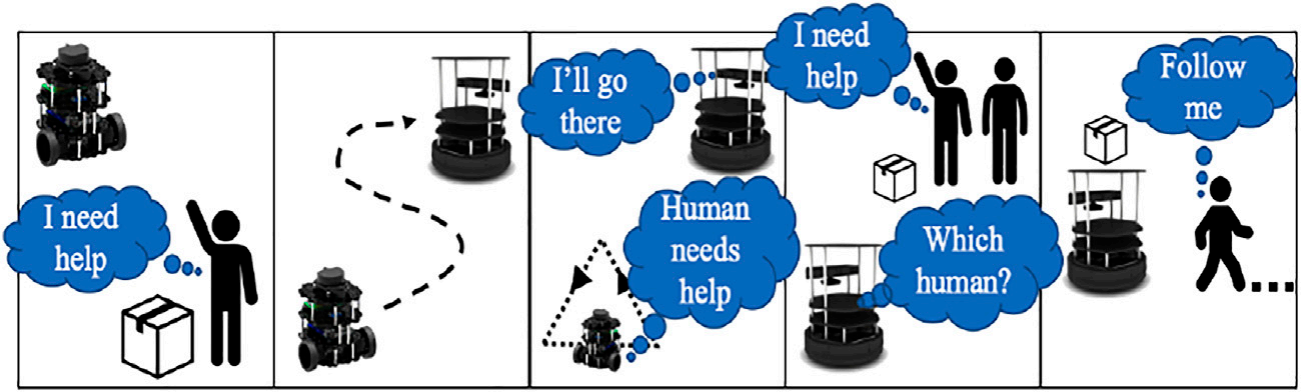
Operational task flow.

The cases for forming different shapes and triangles at different velocities and orientations are carried out in Gazebo. These gestures are tracked with the simulated Kinect camera. The setup is tested on a physical system, and the experimental task flow is depicted in Figure 6.

**FIGURE 7:**
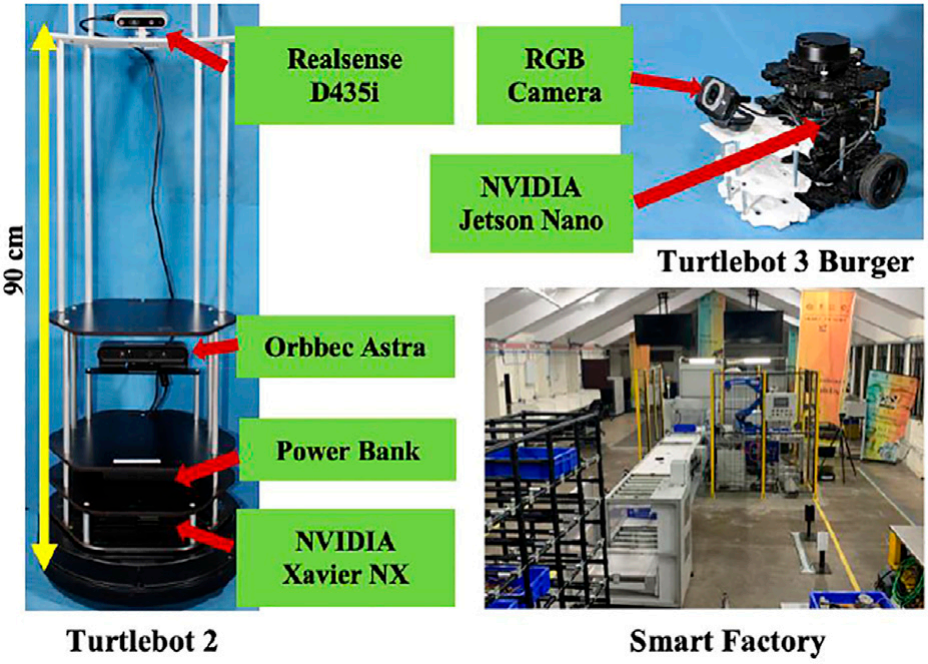
Experimental test set-up.

The setup was tested in an industrial testbed in an area of 15 m × 6 m, consisting of a conveyer belt, a pick-to-light system, and a fixed manipulator, as shown in Figure 7. We test our framework using two modified robot platforms. All the robots were tested on Ubuntu 18.04 running ROS Melodic.

### 4.1 Package Handling Robot

A modified Turtlebot 2 has additional 3D printed plates at heights of 90 and 120 cm to accommodate space for extra sensors and packages. An NVIDIA Xavier NX was mounted on the base of the robot for a physically smaller processing system. Orbbec Astra RGB-D camera is attached at the height of 35 cm for conducting SLAM and Intel Realsense D435i RGB-D camera at the height of 90 cm with a 15° downward tilt for object detection and skeletal tracking. The Realsense cameras are accurate for object tracking and have been used in past literature for tracking position of objects due to their precise depth tracking. It possesses a depth sensor with a resolution upto 1,280 × 720 pixels at 30 frames per second. A brief description of accuracy of object tracking and precision of RealSense cameras has been described and reasoned in (Carfagni et al., 2019).

### 4.2 Messenger Robot

A modified version of a Turtlebot 3 is used for the experiment. Additional 3D printed PLA plates were provided to the robot to accommodate an RGB camera. NVIDIA Jetson Nano was used as an onboard processing system for better image processing and faster computation.

The framework is tested for the following tasks:

i) Messenger robots receive and convey distance and direction of location from human to package handling robots
ii) Messenger robots specify the target package
iii) Instructs package delivery robot to follow messenger robot

Additionally, 3 human operators were working in the given environment in cooperation with the robots. The people were instructed with the gestures and their corresponding commands. Preliminary setup was tested for a given set of velocities and intended directions. For the current setup, the Turtlebot3 (messenger robot) is operated at a maximum velocity v_min_ = 0.05 m/s and the maximum distance in the environment to be d_max_ = 10 m. Subsequently, according to Eq. 8, the proportionality constant k = 0.278. We took certain angles from the range of -150° to 150° at regular intervals at either 15° or 30°. The experiments were initially carried out for the cases of v = 0.05 m/s, 0.08 m/s, 0.1 m/s and 0.2 m/s evaluating to a total number of 64 cases.

## 5 Results

This section analyses the robot-robot interaction framework. The two primary factors to be analyzed were a) How does one robot differentiate between different gestures (shapes) drawn by the other robot? b) What is the accuracy of direction and orientation perceived by the package handling robot? The performance for human gesture identification, object detection, and path planning has not been evaluated since standard libraries and algorithms have been used. The methodologies above have been used only for demonstrating the framework as a whole.

### 5.1 Gesture Formation and Identification

**FIGURE 8:**
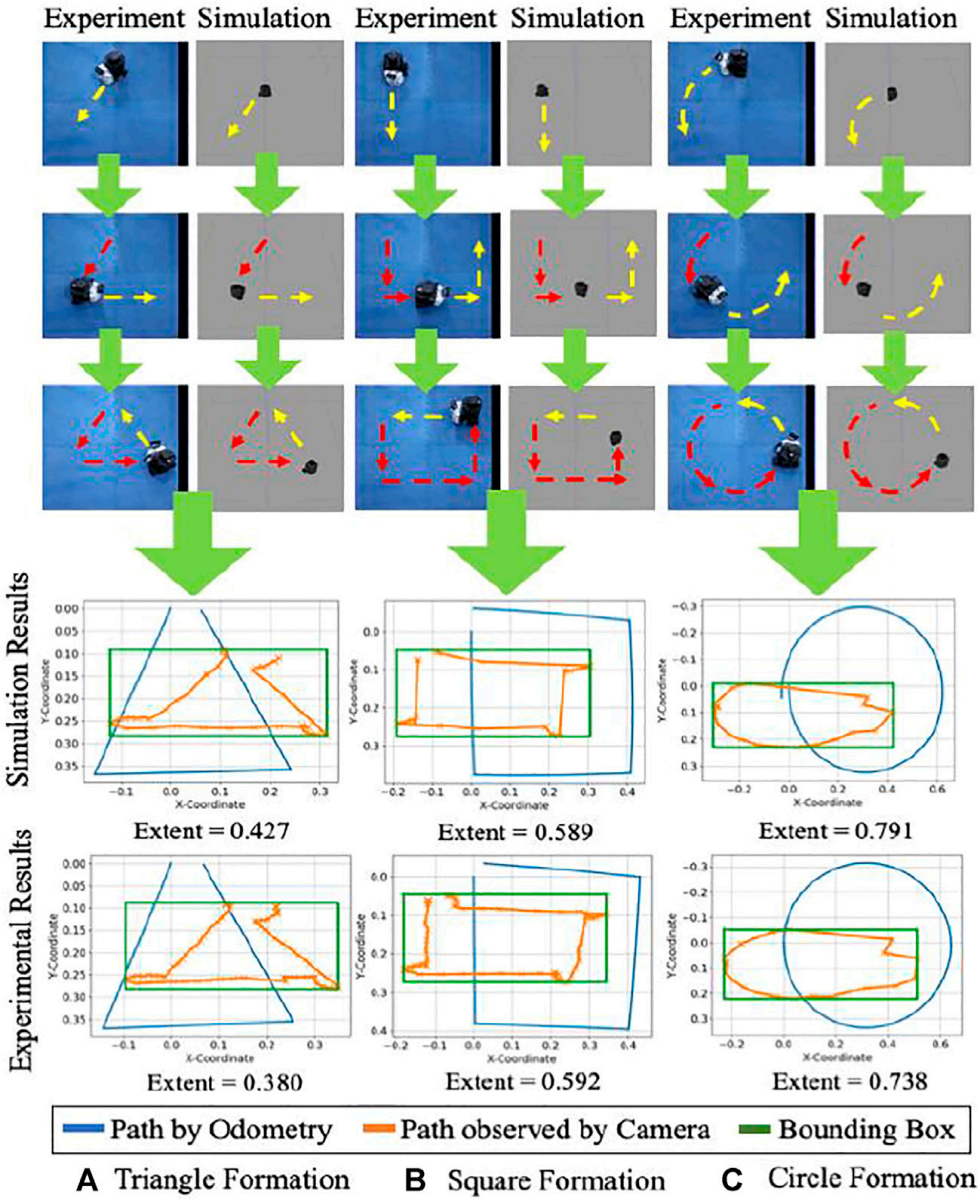
Frame-by-frame shape formation in experiment and simulation. Red arrows indicate the path which has been traced, and yellow arrows indicate the prospective path to be followed. A comparison of odometry data with the path captured by the camera is shown for the physical system and simulation. The bounding box is represented for the observed shape, and the extent of the area is calculated.

Figure 8 describes gesture formation by the messenger robot and identification by the package handling robot. Trials of the messenger robot were conducted for tracing a triangle, square, and circle, according to Eq. 3, the area was calculated for each instance and compared with the given ratio to test for similarity to the shape.

**FIGURE 9:**
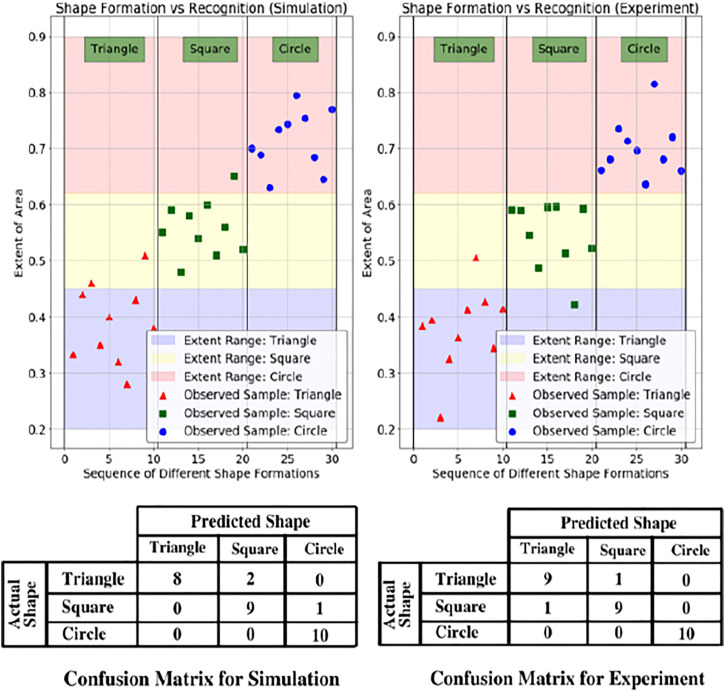
Sample data from simulation and experiment for the extent of area covered and ranges for the corresponding shape. The pink, yellow and purple areas are the empirical regions for the classification of shapes. Below the figure are the confusion matrices for the related scenarios.

An illustration of the same is shown in Figure 8. The values of extent area obtained from these test cases were empirically formulated (Das et al., 2016) to create regions of gesture identification. Triangles were observed to have an extent area ratio of less than 0.45, squares from 0.45 to 0.63, and circles occupying 0.63 to 0.9 part of the bounding box. The same has been depicted for 10 trials, each for a different shape in Figure 9 with a confusion matrix for the number of outliers and inliers. The confusion matrix for the experiments of triangle showed a better result compared to the simulations. This is attributed to a reasoning of a probable better estimate from the camera and tracking system. Moreover, the simulations were carried out in Gazebo which has close similarity to robots simulated in real world environments. Hence, a similar or better confusion matrix can be expected in either the case of simulation or experimental setup.

### 5.2 Gesture Formation and Detection for Different Directions

**FIGURE 10:**
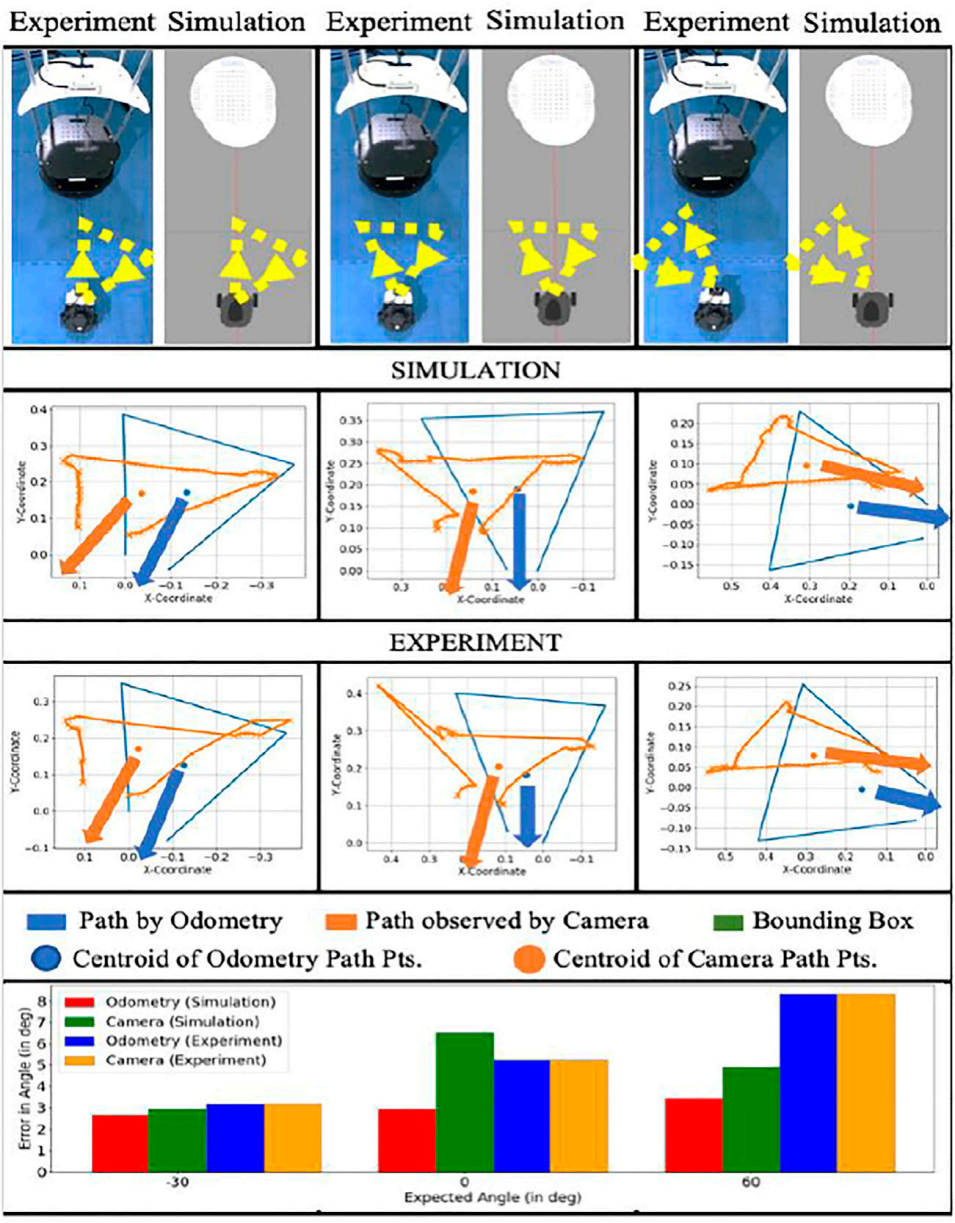
Comparison for few cases of angle orientations and error comparison for odometry and camera perception for simulation and experimental results. The blue and orange arrows indicate the formulated angle. The bar chart depicts the error in the formulated orientation.

**FIGURE 11:**
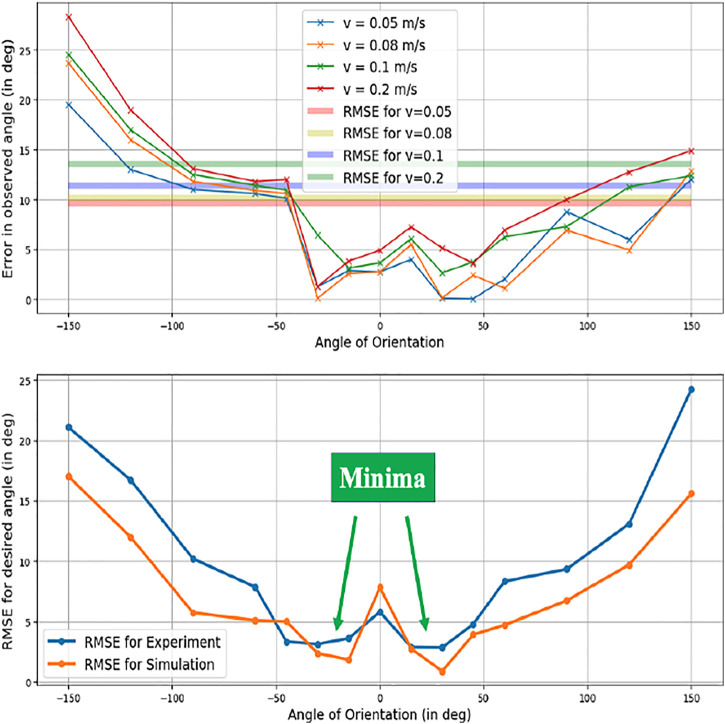
Error plot of directional angles.

**FIGURE 12:**
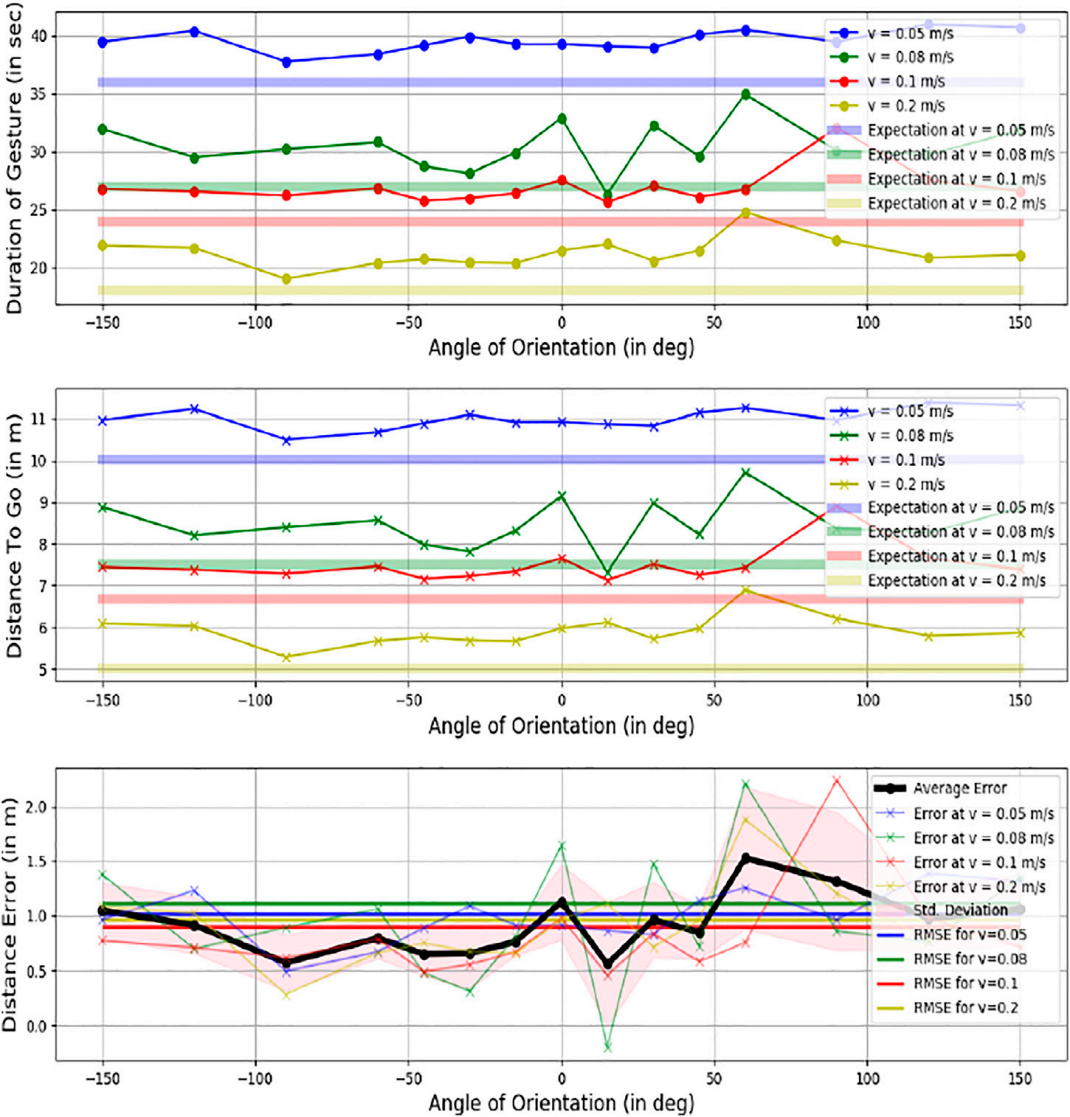
Error plot of distance accuracy.

Figure 10 contains three out of the 64 cases to depict how gestures are identified for different orientations shown by the messenger robot and perceived by the package handling robot. The overall analysis for all 64 cases has been discussed in the results from Figure 11; Figure 12. The vector between the centroid and the midpoint of the average of 5 initial and final points gives the robot’s orientation. The significant error in orientation is due to odometry error which adds up to the error of direction perceived by the camera.

Additionally, trajectory tracking by the camera has multiple fluctuations due to errors in depth estimation by the RGB-D camera, contributing to an added error on top of odometry error.

### 5.2 Performance Evaluation Metrics

#### 5.2.1 Directional Error

Figure 11 describes the error in formulating the intended orientation of the triangle, and subsequently the direction to go in for the 64 cases. The corresponding results are depicted in Figure 12. A minimum error was observed at the angles of -30° and 30°.

$$e_{\theta }=\frac{1}{N}\sqrt{\sum_{i=1}^{N}{\left(\theta_{\mathrm{obs}}-\theta_{\exp}\right)}^{2}}$$ (10)

Where θ_o_ is the observed direction and θ_e_, is the expected direction.

#### 5.2.2 Distance Error

Figure 12 describes the distance accuracy error. This metric analyses the formulation’s efficiency to infer the proper distance to travel for the linear velocity of the robot, i.e., how the error changes as the intended distance are increased or decreased. The duration for each case was calculated based on when the robot starts and stops moving. The corresponding predicted distances (r) were formulated for all angles from Eq. (7). The error in the duration of motion is negligible for simulations since the environment is ideal. The experimental results gave an average error of 1.006 m. It was observed that the error increases as the velocity of robot increases, which can be attributed to the fact that chances of odometry errors are higher at higher speeds

$$e_{r}=\frac{1}{N}\sqrt{\sum_{i=1}^{N}{\left(r_{\mathrm{obs}}-r_{\exp}\right)}^{2}}$$ (11)

#### 5.2.3 Resultant Position Error

**FIGURE 13:**
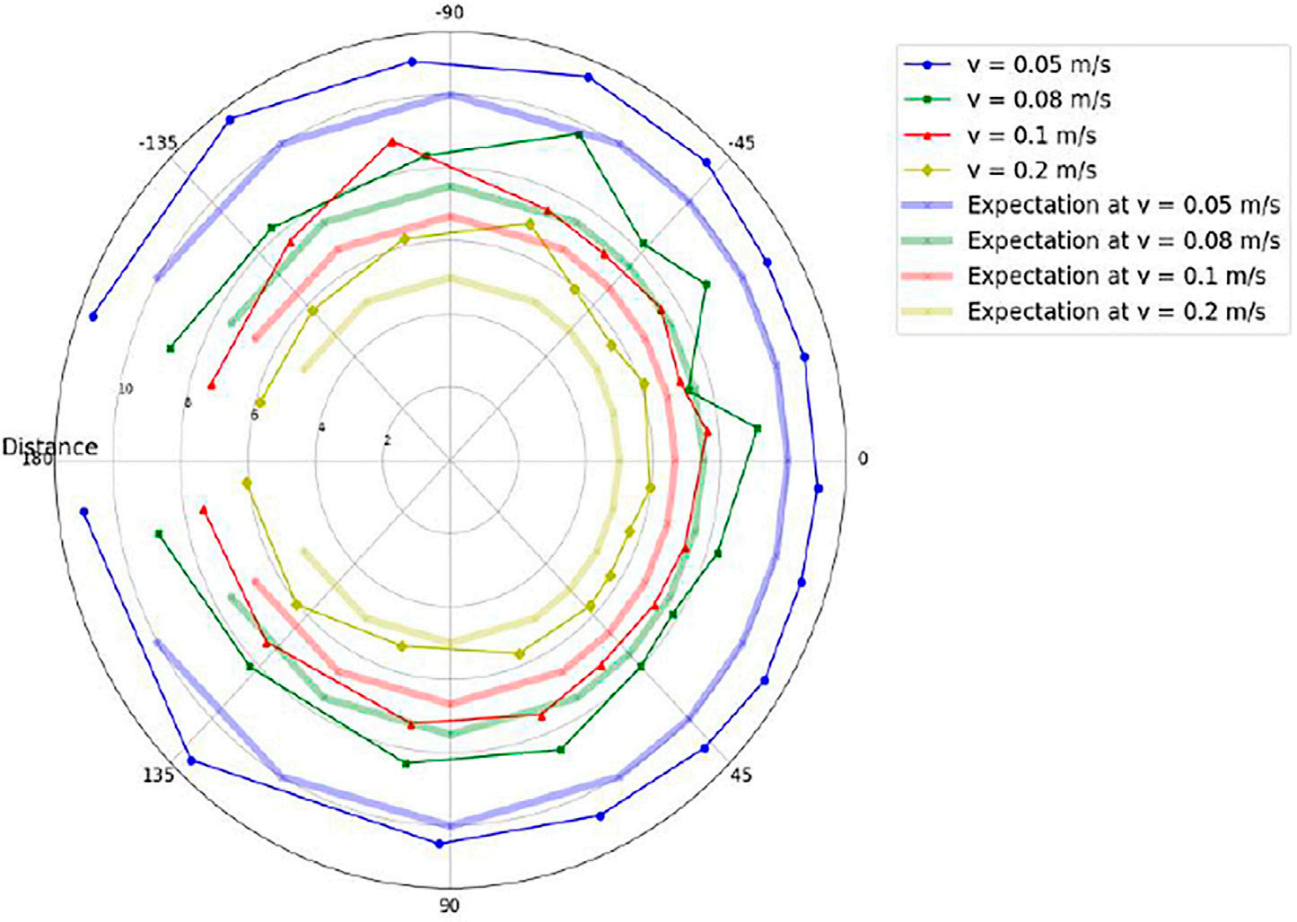
Polar representation of Intended Destination Positions *vs*. Expected Positions.

Here, the overall accuracy is tested by evaluating the final coordinates. Once both distance (r) and direction (θ) values are generated, the resultant vector { $\vec{r}$ ,θ} is formulated. As seen in Table 2, the observed error trend depicts that the error is lower at higher speeds. This shows that the absolute position error is more dependent on the distance error than the orientation error. The resultant coordinates (r, θ) for each case of given orientation and speed are depicted in Figure 9 and the average error is represented in Figure 10. The overall resultant position and orientation of the robot has been represented in Figure 13 as a polar plot. It has been observed that the distance as well as orientation shows a higher error when the speed is higher, or the required orientation is larger. This can also be shown via observations recorded in Table 2. The orientations at −30°and 30 °are the orientations where the messenger robot does not have to take an initial deviation before forming the gesture. Hence, these orientations show the least deviations and errors. Table 2 also draws an interesting perspective that the resultant error is influenced more by the errors observed in the orientation rather than the ones observed in position where ***r*** is a major contributor to the ***e***_***d***_ since error in θ is reduced to a range of [-1,1].

**TABLE 2:** Summary of RMSE.

	e_θ_ (in Degrees)		e_r_ (in m)		e_d_ (in m)	
	Sim.	Exp.	Sim.	Exp.	Sim.	Exp.
0.05	5.295	7.984	∼	1.024	0.391	0.528
0.08	6.378	8.058	0	1.119	0.371	0.468
0.1	7.621	9.572	∼	0.908	0.343	0.432
0.2	8.542	11.915	0	0.972	0.292	0.415
Average	6.959	9.382	∼0	1.006	0.349	0.461

## 6 Conclusion and Future Scope

This paper proposes a novel bio-inspired framework for robots to interact with only visual cues and motion. A cooperative object manipulation task is performed to demonstrate the same by introducing a human-in-the-loop using traditional human-robot interaction methodologies. The key ideas presented in the paper are that a robot can interact with another robot by moving in a particular manner with specific kinematic constraints. Three gestures were demonstrated to signify different commands. Gesture Identification gave an accuracy of 90% for simulations and 93.33% for experiments. A framework for going to a particular location based on a triangle traced by the robot in a specific orientation and time duration was also implemented. The entire framework was tested for its accuracy in conveying the gesture and the intended coordinates in a simulation and an experimental setup. The average positional error was 0.349 m in simulations and 0.461 m for experiments. This framework utilized a team of different robots to demonstrate that the system is scalable. As a future problem, the system can also be trained to recognize the intensity of human gestures. Additionally, the robots can be trained to estimate the trajectory better by modeling a robot after detecting the robot. The applications of this framework in the real world are limitless. As shown in the experimental scenario of the paper, the framework can be used in an industrial environment, but it can also be extended to a search and rescue operation in tight environments where communication may fail. Moreover, it can collaborate with humans and carry out a rescue operation. The main advantage is that it uses only a single modality of vision which allows it to be compatible with robots of various sizes, thereby enabling it to function in a heterogeneous team of robots. The framework can be used to accommodate such multiple robots to create a completely independent and scalable team of robots.

## Data Availability

The raw data supporting the conclusions of this article will be made available by the authors, without undue reservation.
